# Procedural sedation analgesia

**DOI:** 10.4103/1658-354X.62608

**Published:** 2010

**Authors:** Saad A Sheta

**Affiliations:** Oral Maxillofacial Department, Dental College, King Saud University, KSA B.O. 80169 Riyadh 11545, Saudi Arabia

**Keywords:** *Conscious sedation*, *deep sedation*, *minimal anesthesia*, *procedural sedation*

## Abstract

The number of noninvasive and minimally invasive procedures performed outside of the operating room has grown exponentially over the last several decades.Sedation, analgesia, or both may be needed for many of these interventional or diagnostic procedures. Individualized care is important when determining if a patient requires procedural sedation analgesia (PSA). The patient might need an anti-anxiety drug, pain medicine, immobilization, simple reassurance, or a combination of these interventions. The goals of PSA in four different multidisciplinary practices namely; emergency, dentistry, radiology and gastrointestinal endoscopy are discussed in this review article. Some procedures are painful, others painless. Therefore, goals of PSA vary widely. Sedation management can range from minimal sedation, to the extent of minimal anesthesia. Procedural sedation in emergency department (ED) usually requires combinations of multiple agents to reach desired effects of analgesia plus anxiolysis. However, in dental practice, moderate sedation analgesia (known to the dentists as conscious sedation) is usually what is required. It is usually most effective with the combined use of local anesthesia. The mainstay of success for painless imaging is absolute immobility. Immobility can be achieved by deep sedation or minimal anesthesia. On the other hand, moderate sedation, deep sedation, minimal anesthesia and conventional general anesthesia can be all utilized for management of gastrointestinal endoscopy.

## INTRODUCTION

The number of noninvasive and minimally invasive procedures performed outside of the operating room has grown exponentially over the last several decades.[1] Sedation, analgesia, or both may be needed for many of these interventional or diagnostic procedures. With the introduction of shorter-acting sedatives for sedation and opioids for pain control, specific reversal agents for both opioids and benzodiazepines, and the availability of noninvasive monitoring equipment, procedural sedation can now be safely administered in many healthcare settings.[2]

Various procedures that require procedural sedation are better served by considering the goals of procedural sedation and determining if a particular patient requires pharmacological intervention to meet the following goals during a procedure.[3]

- Patient safety;
- Minimizing pain and anxiety associated with procedure;
- Minimizing patient's motion during the procedure;
- Maximizing the chance of success of a procedure; and returning the patient to presedation state as quickly as possible

The indications for procedural sedation can vary from patient to patient, based on anxiety level and pain associated with the procedure[3] [Table 1]. Individualized care is important when determining if a patient requires procedural sedation. The patient might need an anti-anxiety drug, pain medicine, immobilization, simple reassurance, or a combination of these interventions.

**Table 1:** Selected indications for procedural sedation

Dental procedures
Flexible fiber optic laryngoscopy and bronchoscopy
Laceration repair in children
Bone marrow aspiration
Burn debridement/major abrasion cleaning ("road rash")
Cardiac catheterization
Cardio version (elective)
Endoscopy
Fracture reduction/dislocation reduction
Interventional radiology procedures
Thoracentesis
Thoracotomy/chest tube placement

The purpose of this mini-review is to substantially advance our understanding of the goals of PSA in four different multidisciplinary practices namely; emergency, dentistry, radiology and gastrointestinal endoscopy. There, some procedures are painful, others are painless. Therefore, goals of PSA vary widely. Sedation management can range from minimal sedation, to minimal anesthesia.

Sedation and analgesia introduces an independent risk factor for morbidity and mortality in addition to the procedure itself.[4] An anesthetist-led service is ideal but is a scarce resource. Safe protocols and sedation guidelines are beyond the scope of this review because these have been covered extensively elsewhere.[5‐10] Nonetheless, it is mandatory to emphasize that if anesthetists cannot provide a service, then others will need training, support and monitoring, ideally from the local anesthetic department. The Joint Commission on Accreditation of Healthcare Organizations (JCAHO) recognizes the risks involved with sedation and analgesia for procedures and mandates that sedation practices throughout an institution be monitored and evaluated by the department of anesthesia. The American Society of Anesthesiologists (ASA) has responded to this challenging responsibility by developing practice guidelines for non-anesthesiologists who provide sedation and analgesia.[8]

## SEDATION DEFINITIONS

Sedation is a technique where one or more drugs are used to depress the central nervous system of a patient thus reducing the awareness of the patient to his surroundings.

Many professional organizations have published sedation definitions and guidelines, the most prominent being the American Academy of Pediatrics (AAP),[5] the American College of Emergency Physicians (ACEP),[6] the American Society of Anesthesiologists (ASA),[8] and the American Academy of Pediatric Dentistry (AAPD).[7] The ASA revised and updated its guidelines to include a definition of the continuum of sedation that occurs when sedative and analgesic medications are administered.[2,9] From lightest to deepest sedation, the levels are:

Minimal sedation (anxiolysis): In this state, the patient can respond to verbal commands and may have some cognitive impairment, but there is no effect on cardiopulmonary status

Moderate sedation: There is a depression of consciousness, but patients in this state can respond appropriately to verbal commands, either alone or in conjunction with light tactile stimulation. The patient is able to maintain an airway independently, ventilation is adequate, and cardiovascular function is usually unaffected by drugs administered

Deep sedation: Patients in this state are not easily awakened, but they respond purposefully (they do not simply withdraw) after repeated or painful stimulation. These patients may require assistance maintaining an airway and adequate ventilation, but normal cardiovascular status is usually sustained as long as ventilation is appropriate

These revised definitions replace the popular but misused term "conscious sedation," as this level of sedation (as defined by the AAP in 1985)[10] is insufficient for most painful procedures, especially in children.

The term procedural sedation has emerged by The American College of Emergency Physicians (ACEP). Procedural sedation defines as "a technique of administering sedatives or dissociative agents with or without analgesics to induce a state that allows the patient to tolerate unpleasant procedures while maintaining cardio respiratory function. PSA is intended to result in a depressed level of consciousness that allows the patient to maintain oxygenation and airway control independently."[9] Understanding the various depths of sedation is essential to provide safe and effective procedural sedation and analgesia. According to the ASA guidelines, most procedural sedation falls within the level of moderate sedation/analgesia although very painful procedures may require deep sedation/analgesia. The current definition of deep sedation is considered gray. There are, however, important aspects of sedation in uncooperative children that are not covered by the standard definitions and guidelines. With respect to successful painless imaging, the essential element is immobility and, in uncooperative children, the key status is sleep. Therefore, the terms "sleep sedation" or "safe sleep" was evolved and defined as 'the patient is not easily roused but the technique has a safety margin wide enough to render the loss of airway and breathing reflexes unlikely'.[11] Sedation drugs have a wide margin of safety, but are typically weak, and do not always succeed. Occasionally, they cause prolonged unconsciousness usually because of excessive doses. Anesthesia drugs, in contrast, are ideal because they are potent and short acting, and doses can be gradually increased to achieve success. If anesthetic doses are used to induce an unarousable sleep, lasting only a few minutes, subsequent doses could be used that are so low or subanesthetic that, although the individual remains asleep, they may be indeed being rousable. Furthermore, in this state, appreciable effects upon vital reflexes are unlikely and recovery is rapid. The terms 'light anesthesia'[12] or 'minimal anesthesia'[13,14] may be appropriate for such techniques.

## PROCEDURAL SEDATION ANALGESIA IN THE EMERGENCY DEPARTMENT

The success of the physician in management of several painful procedures that may present to emergency department (ED) can be hindered by patient discomfort. Value exists in techniques that allow for alleviating pain and anxiety of the patient undergoing a low-risk procedure while minimizing adverse effects and recovery time; therefore procedural sedation can be a useful tool in emergency department.

Procedural sedation in ED usually requires combinations of multiple agents to reach desired effects of analgesia plus anxiolysis. Various drugs are available to provide procedural sedation.[4] A short-acting benzodiazepine (e.g., midazolam), either alone or in combination with an opioid analgesic (e.g., fentanyl, morphine), is commonly selected for procedural sedation.[15] Evidence in the literature is emerging that also supports the use of other sedatives (e.g., etomidate, propofol) for procedural sedation.[16‐18] Etomidate is gaining popularity because it elicits minimal hemodynamic effects and has a very reliable onset of action[16] .Ketamine results in a dissociative state, and patients may not be able to speak or respond purposefully to verbal commands.[16,18] Ketamine is typically not used in adults because of frequent association with emergence delirium; however, ketamine is used frequently in the pediatric population, where this effect is not typically elicited.[9] The use of propofol and ketamine as single agents for procedural sedation and analgesia in the ED has grown in popularity.[18] The reasonable premise behind ketofol is that the two agents ketamine and propofol are theoretically synergistic. The sympathomimetic properties of ketamine should mitigate propofol-induced hypotension, whereas at the same time propofol might counteract the nauseant and psychic recovery effects of ketamine.[18]

Fospropofol (Aquavan; MGi Pharma, Bloomington, Minn) is a water-soluble prodrug of propofol currently in clinical trials for mild to moderate sedation. However, its clinical use in procedural sedation and analgesia remain to be seen, as it exhibits a longer elimination half-life, larger volume of distribution, and slower onset of action than propofol.[19]

## PROCEDURAL SEDATION ANALGESIA IN DENTISTRY (OFFICE-BASED DENTAL SEDATION)

The main goal of sedation in dentistry is to combat anxiety. The mainstay of the treatment of anxiety is behavioral management. All dentists should be able to communicate well with their patients. If sedation deemed necessary, moderate sedation analgesia (known to the dentists as conscious sedation) is usually what is required.[7,20] It is usually most effective with the combined use of local anesthesia. In dentistry, sedation techniques are not paincontrol techniques and are often overridden when the patient experiences intraoperative pain.[7] To overcome these circumstances with sedative agents alone requires the use of very high doses or the addition of a narcotic to the regimen thus producing deeper levels of sedation than might be required together with the increasing possibility of side effects. Techniques should not be used simply to escape the need to inject a local anesthetic.[7,20]

Dental sedation can be provided in the office sitting, to the patient and he/she allowed home in the same day of surgery and they are commonly performed in a facility away from the proper hospital setting.

With the exception of extensive oral and maxillofacial surgery, most dental procedures are minimally invasive, generally result in little blood loss, and at most elicit pain that can be adequately controlled with oral analgesics.[7] Common indications for office-based sedation are; young children, stressful procedures (such as third molar extraction (most common), complex periodontal procedures, recently dental implants), behaviorally and medically challenged patients.[7]

The use of drugs to help patients deal with their fears has been extensively researched and a number of techniques have become established in dental practice throughout the world. These are, first, oral sedation with benzodiazepines; second, inhalational sedation with nitrous oxide; and third, intravenous sedation with midazolam alone or with an analgesic.[7]

A number of more innovative sedation techniques have been investigated in recent years, including polypharmacy,[21]intravenous sedation in children,[22,23] inhalational sedation with sevoflurane,[24,25] trans-mucosal or intranasal sedation,[26] intravenous sedation with propofol and dental sedation with dexmedetomidine.[27] New concepts in sedation for dentistry include enhanced mechanisms for drug delivery such as target controlled minfusion (TCI) and Patient controlled sedation (PCS).[28]

## PROCEDURAL SEDATION ANALGESIA FOR GASTROINTESTINAL ENDOSCOPY

Goals of procedural sedation for management of gastrointestinal endoscopy vary from one case to another. Moderate sedation, deep sedation, minimal anesthesia and conventional general anesthesia can all be utilized.

Moderate sedation is possible[29] again, if the child is not cooperative, deeper sedation is necessary. Combinations of midazolam, fentanyl and ketamine are useful but must be used with caution.[30] Even with good judgment, there is a risk of laryngospasm[31] and hypoxia.[32] Recovery is usually prolonged and naloxone and flumazenil are often needed.[29]

General anesthesia, favored by anesthetists, has been shown to provide safer operating conditions than sedation.[33] but is perceived, by endoscopists, as being expensive because it requires specialized personnel.[32] However, the short-acting nature of propofol[34,35] and sevoflurane.[36] can reduce costs because recovery time is shorter. Anesthesia avoids the considerable expense of sedation failure. Colonoscopy causes pain from bowel distension, which may serve to warn of colonic perforation. Anesthesia reduces colonic tone and therefore deep sedation may be safer in this respect.

Minimal anesthesia with an initial bolus of propofol (2-3 mg/kg)) will suppress the gag reflex in upper gastrointestinal endoscopy sufficiently for insertion of the scope and smaller bolus doses or an infusion are effective thereafter.[35,37]Supplementary opioids are not usually necessary. Anesthesia for upper endoscopy may not require tracheal intubation as diagnostic procedures can take less than 10 min and do not cause appreciable unpleasant after effects. Two safety points need emphasis: the endoscope can compress and obstruct the trachea (especially in infants)[37,38] and achalasia is very dangerous (the esophageal residue should be drained before any sedation or anesthesia is given). Tracheal intubation is much safer in these two situations. Also anesthesia for endoscopy, lasting 15-30 min, may be easier with an airway device; facemasks or laryngeal masks are available that have a port for the endoscope.[39]

In literature, reports discussed providing minimal anesthesia in gastrointestinal endoscopy are mostly managed by non-anesthetists. Readers should note that few are published in the anesthesia literature. Anesthetists are naturally concerned about the safety of a technique in which gastric contents could potentially be aspirated and also about whether non-anesthetists have sufficient judgment and skill to use propofol.

## PROCEDURAL SEDATION ANALGESIA FOR PAINLESS IMAGING

The mainstay of success for painless imaging is absolute immobility. Immobility can be achieved by either deep sedation or minimal anesthesia.

Natural sleep is only sufficiently reliable in infants under 6 months old who will often sleep after a feed and if they are warm (the 'feed and wrap' technique).[13]Older children may sleep naturally after melatonin, but this is probably not reliable enough to be practical.[40]

Regardless of potential side effects,[41]large doses of oral chloral hydrate or triclofos (50- 100 mg/kg, maximum dose 1 g) reliably causes sleep lasting 30-60 minutes in 95% of children below 15 kg and are effective for MR imaging.[42]Deep sedation for uncooperative children over 15 kg can be difficult. The volume of chloral required often causes vomiting and benzodiazepines alone are usually insufficient for scans that need prolonged sleep. Dexmedetomidine shows promise because it seems to preserve rousability. It has to be infused intravenously and has been combined with either midazolam[43]or chloral.[44]

Propofol is the ideal intravenous 'minimal' anesthetic. After a standard induction dose, maintains an immobile sleep in almost all children.[14] Appreciable airway effects occur in 1-2% but these should only need simple support measures. Occasionally, propofol alone does not suppress involuntary movements,[45] but a recent report confirms that propofol does not trigger epileptiform cortical electric activity in epileptic children.[46]

Pentobarbital (up to 5 mg/k) is regarded as a safe intravenous sedative in North America; however, significant numbers of children have airway obstruction or paradoxical excitement.[47] Thiopentone is a reliable drug for short scans.[48] Sevoflurane and other vapors can be given so that, after induction, the children can be positioned so that airway support is unnecessary.[13,14]Control of inspired concentrations is imprecise, but endtidal concentrations guide vaporizer settings. Scavenging could be achieved via a transparent plastic hood. Ketamine can cause involuntary movements that spoil the images; it also causes vomiting and distressing hallucinations.[ 13,14]
